# Editorial: Novel Aspects of Nucleolar Functions in Plant Growth and Development

**DOI:** 10.3389/fpls.2018.00814

**Published:** 2018-06-14

**Authors:** Munetaka Sugiyama, Yasunori Machida

**Affiliations:** ^1^Botanical Gardens, Graduate School of Science, The University of Tokyo, Tokyo, Japan; ^2^Division of Biological Science, Graduate School of Science, Nagoya University, Nagoya, Japan

**Keywords:** nucleolus, ribosome biogenesis, plant, growth, development, environmental response

The nucleolus is a prominent nuclear body that is common to eukaryotes. Since the nucleolus was first described in the 1830s, its identity had remained a mystery for longer than 100 years. Major advances in understanding of the nucleolus were achieved through electron microscopic and biochemical studies in the 1960s to 1970s followed by molecular biological studies. These studies finally established the view of the nucleolus that it is a large aggregate of RNA-protein complexes associated with the rRNA gene region of chromosome DNA, serving mainly as a site of ribosome biogenesis, where pre-rRNA transcription, pre-rRNA processing, and assembly of rRNAs and ribosomal proteins (r-proteins) into ribosome subunits occur. This function of the nucleolus appears to indicate that the nucleolus plays a constitutive and essential role in fundamental cellular activities by producing ribosomes. Recent research has shown, however, that the nucleolus is more dynamic and can have more specific and wider functions.

In plants, nucleolar functions have been lately implicated in developmental regulations and environmental responses from experimental evidence obtained mostly from genetic studies of nucleolus-related mutants. Detailed and comprehensive analysis of nucleolar components and molecular cytological characterization of sub-nucleolar domains have also provided new insights into functions and behaviors of the plant nucleolus. This Research Topic has collected articles concerning recent findings from the plant nucleolar research, with a primary focus on physiological and molecular links of the nucleolus to growth and development in plants.

In the model plant Arabidopsis, a number of mutants have been reported for genes encoding r-proteins and ribosome biogenesis factors (RBFs). Previous works showed that most of these mutants share developmental phenotypes, one of the most typical examples of which is a pointed leaf shape. Many of these mutations are also known to affect leaf polarity and cause severe leaf abaxialization in a sensitized genetic background such as *asymmetric leaves2* (*as2*). Kojima et al. characterized two pointed-leaf mutants, *oligocellula2* (*oli2*) and *g-patch domain protein1* (*gdp1*) and demonstrated that these mutations synergistically repress cell proliferation in leaf primordia and that either of them do not strongly enhance leaf abaxialization in *as2*. Both OLI2 and GDP1 proteins were shown to localize in the nucleolus and participate in ribosome biogenesis. These results suggest that the leaf cell proliferation defect and leaf abaxialization triggered by mutations in r-protein genes or RBF genes may be mediated by different mechanisms.

In animals, it is well known that perturbations of ribosome biogenesis in the nucleolus cause a particular type of stress called nucleolar stress (or ribosomal stress) activating specific signaling pathways to induce cell cycle arrest or apoptosis. In plants, however, a corresponding stress response pathway had long been unrecognized. Very recently, loss-of-function mutations in the NAC transcription factor gene *ANAC082* were found to suppress growth defects and developmental abnormalities in various ribosome biogenesis-impaired mutants, which has led to the hypothesis that plants respond to nucleolar stress via a plant-unique, ANAC082-dependent signaling pathway. Ohbayashi and Sugiyama outline these studies along with their research background and introduce the concept of plant nucleolar stress response as a new face of NAC-dependent cellular stress responses.

Phenotypic studies of mutants impaired in genes encoding r-proteins or RBFs have also indicated important roles of the nucleolus in various environmental responses in plants. Liu and Imai present a mini-review focusing on DExD/U-box RNA helicases among RBFs, in which information about plant DExD/U-box RNA helicases with functions in ribosome biogenesis is surveyed to highlight their involvement in adaptation to environmental stresses such as high and low temperatures.

Molecular biological reexamination of nucleolar components is another trend in exploring the hidden functions of the plant nucleolus. Fibrillarin is a major conserved nucleolar protein, which localizes at the boundary between fibrillar center and fibrillar component of the nucleolus as well as in the nucleolus-related nuclear domain Cajal body and is considered to act as a methyltransferase in the initial stage of pre-rRNA processing. Notably, most of plants have two or more different fibrillarin genes. Rodriguez-Corona et al. investigated two Arabidopsis fibrillarins AtFib1 and AtFib2 and detected a novel ribonuclease activity only in AtFib2. This discovery suggests an unidentified role of plant fibrillarin.

Fibrillarin and several other nucleolar components have been found to associate with pathogen-derived factors such as plant viral proteins, and today the role of the nucleolus in plant-pathogen interaction has become an important topic. With a special emphasis on this aspect, Kalinina et al. summarize a broad range of findings to depict a comprehensive view of the multifaceted functions of the nucleolus in growth, development, disease, and stress responses of plants.

Nucleolar proteome analysis has contributed very much to our understanding of the nucleolus. Montacié et al. carried out proteome analysis of highly purified nucleoli of Arabidopsis by mass spectrometry and identified many RBFs and also proteins non-related to ribosome biogenesis, which interestingly contain proteins of 26S proteasome. By further experiments, they demonstrated the nucleolar localization of 26S proteasome subunits and an interplay between proteasome activity and nucleolar organization.

Small nuclear ribonucleoproteins (snRNPs), complexes of proteins and a specific class of non-coding RNAs involved in RNA processing events such as pre-mRNA splicing, are contained not only in the nucleoplasm but also in the nucleolus and Cajal bodies. It is now considered that critical steps of snRNP biogenesis occur in the nucleolus and Cajal bodies. Ohtani provides an overview of the current knowledge about the roles of the nucleolus and Cajal bodies in snRNA biogenesis and discusses its possible relation to plant development and environmental responses.

Imaging techniques are nowadays essential in molecular cell biology research. Labeling of RNA with 5′-ethynyl uridine (EU) is one of such techniques recently established. Dvořáčková and Fajkus present a protocol of EU labeling optimized for visualization of plant nucleoli, which is very useful for studying nucelolar behavior and activities in plant cells.

We hope that these articles arouse interest in expanding aspects of the nucleolar functions in plants beyond the classical view of the nucleolus and inspire new research on the nucleolus across various fields of plant science.

## Author contributions

MS wrote the draft and YM edited it. MS and YM revised the manuscript.

### Conflict of interest statement

The authors declare that the research was conducted in the absence of any commercial or financial relationships that could be construed as a potential conflict of interest.

